# Editorial: Omics techniques in deciphering environmental, industrial and therapeutic applications of microbes

**DOI:** 10.3389/fmicb.2023.1327368

**Published:** 2023-11-28

**Authors:** Utkarsh Sood, Janmejay Pandey, Sanjay Kumar Singh Patel, Helianthous Verma

**Affiliations:** ^1^Department of Zoology, Kirori Mal College, University of Delhi, New Delhi, India; ^2^Department of Biotechnology, Central University of Rajasthan, Ajmer, India; ^3^Department of Chemical Engineering, Konkuk University, Seoul, Republic of Korea; ^4^Departmet of Zoology, Ramjas College, University of Delhi, New Delhi, India

**Keywords:** Omics, genomics, metagenomics, metaproteomics, biotechnologic application, therapeutic, environmental microbe

Microbes are essential for life to exist on Earth. These tiny organisms play an important role in the sustainability of the environment and human health. They produce a large number of biotechnologically important products and also certain metabolites that can affect humans in both positive and negative ways. Omics techniques such as genomics, transcriptomics, proteomics and metabolomics, have revolutionized our understanding of microbes and their role in environment, industrial and therapeutic applications. These techniques let investigators analyze the microbe's genetic, protein, and metabolic profiles and provide valuable insights into their responses to environmental factors, biotechnological potential and their functional interactions.

The Research Topic aims to highlight the application of omic techniques in deciphering the environmental, industrial and therapeutic application of microbes. Nine articles have been published on this topic, which provide insights into the diverse roles that microbes play in different settings.

Sharma et al. reported a novel hyperactive (142 μg/mL/min) and thermostable keratinase from *Bacillus pacificus* RSA27 for the valorization of chicken feather waste to peptides rich in essential amino acids. *In silico* analysis on the protein sequence of the keratinase validated the high-affinity calcium-binding site (Asp128, Leu162, Asn164, Ile166, and Val168) and a catalytic triad of Asp119, His151, and Ser308 of keratinase belongs to serine protease. The biotechnologically important product was scaled to 5L fermeter, and it was to achieve ~94% hydrolysis with a total of 154 μmol/mL amino acids production without any cytotoxic influence proving its broad biotechnological applications potential.

Bilinski et al. used next-generation sequencing microbes culturing methods (aerobic and anaerobic conditions) and flow cytometry (viability measurements), for the assessments of fecal microbiota transplantation as fresh vs. frozen stool without any cryoprotectants. They reported that freezing stool samples enormously impacted cultivable bacterial community structure, evidenced by a significant drop in *Actinobacteria* and *Bacilli* that was also confirmed using the amplicon sequencing.

Genomics plays a pivotal role in uncovering the biotechnological potential of microbes by revealing their genetic makeup and functional capabilities, aiding in the identification of valuable genes for applications in medicine, agriculture, industry, and environmental remediation. It allows for the targeted manipulation and optimization of microbial strains for enhanced production of biofuels, biopharmaceuticals, enzymes, and other bioproducts. Yadav et al. isolated and characterized a human intestinal bacterium *Cytobacillus oceanisediminis* NB2 and subjected it to whole genome sequencing. The genomic analysis of this strain identified the presence of gene clusters for diverse bio-catalytic activity, stress response, and antimicrobial activity, as well as indicated the absence of pathogenic gene islands. The strain being native to the human gut also displayed probiotic potential as its genetic repertoire coded for functional features like anti-amylase, anti-lipase, glutenase, prolyl endopeptidase, lactase, bile salt hydrolase, cholesterol oxidase and anti-pathogenic activity.

Gattoni et al. isolated a novel low-abundant rare biosphere marine bacterium *Leeuwenhoekiella parthenopeia* sp. nov. Mr9^T^ and sequenced its complete genome. The genome sequence validated the strain Mr9^T^ was a novel species of the phylum Bacteroidota. The genome was also mapped to the Mediterranean Sea metagenomes revealing an abundance of ~0.003% of the bacterial population in the metagenomes. The genomic information of the strain revealed the presence of five biosynthetic gene clusters with biotechnological applications. The total lipid content (lipidome) was found to inhibit tumor cell viability.

Ngamcharungchit et al. isolated a new marine actinomycete, strain MCN248 from coastal sediment in Thailand. It shared genetic similarities with *Nonomuraea harbinensis* and *Nonomuraea ferruginea* but exhibited low DNA-DNA hybridization relatedness. Genomic analysis revealed the potential for biosynthesis of various secondary metabolites, including anticancer compounds. The crude extract from this strain inhibited colorectal cancer cells.

Metagenomics, which focuses on analyzing the ecological sample's genetic pool, has been significantly used to investigate microbial communities' diversity and functional capacity. It has uncovered the microbial communities' vast genomic variety and their roles in the ecosystem, such as nutrient cycling and pollutants degradation. Nimonkar et al. reported that oligotrophs and copiotrophic microorganisms at a hypersaline and hyper-alkaline Lonar Lake (Maharashtra, India). Further, they showed that compositional changes in the culture media led to a significant alteration in the selection of organisms within the same sample. Isolated oligotrophs with valuable enzyme production potential can be a valuable resource for cost-effective industrial enzyme production. Additionally, these oligotrophs can serve as a tool for OMICS studies to gain insights into how microbes adapt and survive in environments with very limited nutritional resources, and this knowledge can be applied to similar situations.

The study by Wing-Tung Lee et al. investigated microbial communities in the built environment, aiming to improve the assessment of pathogenic risks beyond traditional bacterial culture methods. The research utilizes 16S rRNA gene analysis with nanopore sequencing, comparing various taxonomic classifiers (ARGpore2, Emu, Kraken2/Bracken, and NanoCLUST) in terms of their performance. The results indicate that NanoCLUST is a preferred option for microbial profiling, displaying high concordance with dominant species and similar profiles to MegaBLAST. For identifying culturable species, Emu demonstrates the highest accuracy (81.2%) and F1 score (29%). This research contributes valuable insights for future microbial community studies, particularly those employing nanopore 16S rRNA sequencing in complex environments.

Zhang et al. reported the genomic diversity and characteristics of Monascus species, which are significant in the food industry but can also produce citrinin mycotoxin. Through genomic analyses and comparative genomics, they identified two major clades within the *Monascus purpureus* clade and the *M. pilosus-M. ruber* clade, with *M. pilosus* and *M. ruber* being closely related. The research highlights differences in gene content related to environmental adaptation and reveals gene clusters for pigment synthesis and citrinin production. Notably, the citrinin gene cluster is found only in *M. purpureus*, while the monacolin K gene cluster is present in *M. pilosus* and *M. ruber*, with variations in sequence conservation. This study enhances our understanding of Monascus species in terms of classification, metabolism, and safety, providing valuable insights for further research in the field.

Metaproteomics, an essential field in microbiome research, characterizes microbial community proteomes. The study by Ascandari et al. employed bibliometric analysis to assess the global landscape of metaproteomic research and Africa's contribution. The number of metaproteomic publications has risen significantly, with notable contributions from the USA, Germany, China, and Canada. Frontiers in Microbiology is a key publishing platform. While Africa's contribution is limited (2.2% of total publications), South Africa stands out. Importantly, more than half of Africa's publications have a high Field-Weighted Citation Impact (FWCI), indicating their significance. To enhance Africa's involvement in this field, investments, collaborations, and mentorship programs are vital for future progress.

Articles published in the Research Topic, hence, are of wide “Omics” disciplines which holds valuable information on microbes and their ability to produce biotechnological, therapeutical and industrially important compounds. Also, microbial adaptations to the environment with minimal nutrient requirement for cost effective enzyme production and better methods such as NanoCLUST for assessment beyond the traditional method has been reported in the Research Topic. Thus, these studies enhanced the knowledge of bacterial compounds and the OMICs methods which can further be exploted to obtain useful information in exploring microbial world.

## Author contributions

HV: Writing – original draft, Writing – review & editing. US: Writing – original draft. JP: Writing – review & editing. SP: Writing – review & editing.

